# Evaluation of Antioxidant and Enzyme Inhibition Properties of Siran Propolis: Correlations With Phenolic Content Determined by LC–MS/MS

**DOI:** 10.1002/fsn3.70654

**Published:** 2025-07-21

**Authors:** Neslihan Balci, Kubra Aslan, Adem Erturk, Ilhami Gulcin

**Affiliations:** ^1^ Dursun Keles Health Services Vocational School Gumushane University Gumushane Türkiye; ^2^ Faculty of Science, Chemistry Department Ataturk University Erzurum Türkiye; ^3^ Rectorate of Agri Ibrahim Cecen University Agri Türkiye

**Keywords:** antioxidant activity, enzyme inhibition, LC–MS/MS analysis, propolis

## Abstract

This study evaluates the antioxidant activity, enzyme inhibition potential, and phenolic composition of ethanolic Siran propolis extract. Antioxidant capacity was measured using reducing power (CUPRAC, FRAP, and Fe^3+^‐reducing) and radical scavenging (DPPH·, ABTS·^+^, and DMPD·^+^) assays. The extract showed strong reducing ability, surpassing standard antioxidants in CUPRAC and FRAP assays, correlating with high ferulic acid (40.40 mg/g) and p‐coumaric acid (88.60 mg/g) levels identified by LC–MS/MS. While it demonstrated notable DPPH· scavenging (IC_50_: 30.14 μg/mL), ABTS^·+^ activity was weaker (IC_50_: 272.44 μg/mL). Enzyme inhibition assays indicated effective inhibition of α‐glycosidase, AChE (IC_50_: 2.15 μg/mL), hCA I, and hCA II. Notably, AChE inhibition was stronger than the standard tacrine inhibitor (IC_50_: 8.82 μg/mL), suggesting neuroprotective potential. The observed bioactivities are attributed to synergistic effects of phenolic acids and flavonoids, including quercetin, luteolin, taxifolin, and resveratrol. These findings highlight Siran propolis as a promising natural antioxidant and enzyme inhibitor source with potential therapeutic applications and food formulations.

## Graphical abstract

The ethanolic extract of Şiran propolis exhibited strong antioxidant activity and inhibited α‐glycosidase, AChE, and human carbonic anhydrase isozymes I and II. High levels of phenolic acids, especially p‐coumaric and ferulic acids, correlated with its bioactivity. These results suggest Şiran propolis as a potential natural source for therapeutic and functional food applications.

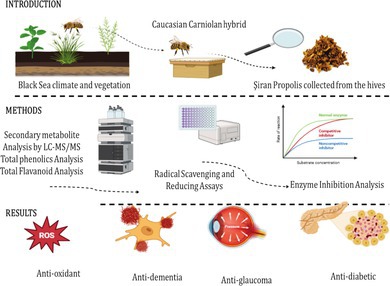

**Abbreviations**
ABTS·+: 2,2′‐Azino‐bis(3‐ethylbenzothiazoline‐6‐sulfonic acid) radical cation
AChE: Acetylcholinesterase
CUPRAC: Cupric Ion Reducing Antioxidant Capacity
DMPD·+: N,N‐Dimethyl‐p‐phenylenediamine radical cation
DPPH·: 1,1‐Diphenyl‐2‐picrylhydrazyl radical
FRAP: Ferric Reducing Antioxidant Power
GAE: Gallic Acid Equivalent
hCA I/II: Human Carbonic Anhydrase I/II isoenzymes
IC_50_
: Half maximal inhibitory concentration
LC–MS/MS: Liquid Chromatography–Tandem Mass Spectrometry
PBS: Phosphate Buffered Saline
QE: Quercetin Equivalent
TFC: Total Flavonoid Content
TPC: Total Phenolic Content

## 1 Introduction

Propolis is a sticky, gummy, and balsamic substance produced by honeybees ( *Apis mellifera* L.) through the collection of resins from various plants. Bees use propolis to coat their hives, protecting them from diseases caused by microorganisms and predators sharing the same habitat (Hossain et al. 2022). Throughout history, both bees and propolis have played important roles in traditional medicine and daily life across diverse cultures, including the Egyptians, Georgians, Greeks, and Romans. For example, the Egyptians used propolis in mummification, while Georgians applied propolis ointments to heal wounds and relieve pain (El‐Guendouz et al. 2019; Hossain et al. 2022).

Additionally, various propolis‐based formulations—such as sprays, ointments, and powders (primarily comprising tinctures and ethanolic extracts)—were developed to treat colds, flu, bronchial asthma, and other ailments (Hossain et al. 2022; Matuszewska et al. 2021). For years, ethanolic extracts of propolis have been recognized for their anti‐inflammatory properties and used as immunomodulatory agents (Matuszewska et al. 2021; Wagh 2013). Today, propolis continues to be used in treating wounds, burns, sore throats, dental caries, and stomach ulcers, and it shows promise in dentistry for managing infections in endodontics (Ahangari et al. 2018; Hossain et al. 2022; Matuszewska et al. 2021; Wagh 2013). Moreover, it is widely employed as an immunostimulant to help prevent colds due to its antibacterial and antiviral actions, as well as a natural remedy for skin problems, small oral ulcers, and urinary tract discomfort, and to restore the balance of the gastric mucosa (Ahangari et al. 2018; El‐Guendouz et al. 2019; Hossain et al. 2022; Wagh 2013).

Building on the extensive historical use of propolis in traditional medicine, propolis has become known all around the world. Chemical composition, biological, and pharmacological effects of the propolis have been discovered and revealed in the studies (Altieri et al. 2024; Belmehdi et al. 2023; Böke Sarıkahya et al. 2021; El‐Guendouz et al. 2019; Gülçin et al. 2010; Karagecili, Yılmaz, et al. 2023; Wagh 2013). According to the studies, the main component of propolis is plant resins; however, many other compounds, including waxes, essential oils, pollens, and other organic substances in different percentages, were also identified in the different types of extracts (Ahangari et al. 2018; Belmehdi et al. 2023; Hossain et al. 2022). Many studies reported that propolis contains, in particular, phenolic acids, flavonoids, ketones, aldehydes, chalcones, dihydrochalcones, terpenoids, amino acids, aliphatic acids, aromatic esters and acids, carbohydrates, vitamins, metals, and also beeswax (Ahangari et al. 2018; Belmehdi et al. 2023; El‐Guendouz et al. 2019; Gülçin et al. 2010; Hossain et al. 2022; Karagecili, Yılmaz, et al. 2023; Matuszewska et al. 2021; Wagh 2013).

The precise composition of propolis varies according to plant source, season of harvesting, geography, type of bee flora, climate changes, and honeybee species at the site of collection (Belmehdi et al. 2023; Hossain et al. 2022; Karagecili, Yılmaz, et al. 2023; Matuszewska et al. 2021). In general, there have been attempts to classify samples from around the globe according to their chemical composition and floral origin. Poplar (temperate), Birch, Tropical, Mediterranean, and Pacific are the main recognized classes of propolis. Propolis from temperate regions—including West Asia, North Africa, Europe, North America, parts of Argentina, and New Zealand—exhibits a relatively uniform phytochemical profile, predominantly consisting of flavonoids and phenolic acid esters. In contrast, these compounds are nearly absent in tropical propolis, which is instead rich in polyprenylated benzophenones and lignans. Samples from Russia, as well as mountainous regions of Switzerland and Italy, are characterized by phenolic glycerides, such as dicoumaroyl acetyl‐, diferuloyl acetyl‐, feruloyl coumaroyl acetyl‐, and caffeoyl coumaroyl acetyl glycerol. Meanwhile, Mediterranean propolis—from countries like Croatia, Algeria, Greece (Evia Island), and Cyprus—features a distinct composition marked by high concentrations of diterpenoids and minimal or negligible amounts of flavonoids. Lastly, Pacific propolis, collected from Taiwan and Japan (Okinawa), primarily comprises prenylated flavanones (Kasote et al. 2022).

Despite the well‐documented bioactivity of propolis from various regions, the chemical composition and therapeutic potential of propolis from Şiran, Türkiye, remain largely unexplored. Given Şiran's rich biodiversity, unique floristic composition, mountainous terrain, and low levels of industrial activity, the region offers a promising yet underexplored source of propolis with potentially distinct phytochemical and pharmacological characteristics. This study analyzed propolis collected from Şiran using LC–MS/MS and spectrophotometric methods to determine its phytochemical composition. Additionally, its biological activities were assessed through in vitro antioxidant assays and enzyme inhibition studies targeting key enzymes involved in metabolic disorders. These findings may contribute to a deeper understanding of Şiran propolis and its potential role in therapeutic applications.

## 2 Material and Methods

### 2.1 Chemicals

N, N‐Dimethyl‐p‐phenylenediamine dihydrochloride (DMPD), 1,1‐diphenyl‐2‐picrylhydrazyl (DPPH), 2,2′‐azino‐bis 3‐ethylbenzthiazoline‐6‐sulfonic acid (ABTS), neocuproine (2,9‐dimethyl‐1,10‐phenanthroline), ascorbic acid, butylated hydroxytoluene (BHT), butylated hydroxyanisole (BHA), α‐tocopherol, Trolox, Folin Ciocalteu's and Ellman's reagents (5,5′‐dithio‐*bis*‐(2‐nitrobenzoic acid)) were purchased from Sigma‐Aldrich (GmbH, Steinheim, Germany). Standard phenolics used in LC/MS–MS calibration and validation were purchased from Sigma Aldrich GmbH (Steinheim, Germany). Acetylcholinesterase (AChE) from *Electrophorus electricus* (electric eel), Type VI‐S lyophilized powder 200–1000 U/mg protein, Butyrylcholinesterase (BChE) from equine serum, lyophilized powder ≥ 900 units/mg protein, Alpha‐Glycosidase Type I from Baker's Yeast 100 UN were purchased from Sigma Aldrich GmbH. Human carbonic anhydrase I and II isoenzymes (hCA I and hCA II) were isolated from human erythrocytes through hemolysis, purified by affinity column chromatography, and characterized by SDS‐PAGE as previously applied (Beydemir and Gülçin 2004; Taslimi et al. 2016). Acetylthiocholine iodide 99.0%, Butyrylthiocholine iodide ≥ 98%, 5,5′‐Dithiobis (2‐Nitrobenzoic Acid), 4‐Nitrophenyl β‐D‐glucopyranoside (p‐NPG) ≥ 98% (TLC), and 4‐Nitrophenyl acetate (p‐NFA) ≥ 99.0% (GC) were purchased from Sigma Aldrich. Other chemicals used but not mentioned are of analytical grade.

### 2.2 Preparation of Propolis

Propolis was collected from Yukarı Kulaca Köyü (1600–3331 m altitude), a village located in the Şiran district of Gümüşhane Province, Türkiye (40.075° N latitude and 39.115° E longitude). The hives belonged to the Caucasian Carniolan hybrid bees, and the majority of the plants in the area were thyme, mint, and astragalus. Ethanolic extract of propolis was prepared by three times extraction of equal volumes of ethanol and propolis. The organic layer was then collected and evaporated under dryness at 50°C using a rotary evaporator (Heidolph Hei‐Vap Precision G3 Rotary Evaporator, Schwabach, Germany).

### 2.3 Determination of Phytochemistry of Propolis

#### 2.3.1 Total Phenolic Content (TPC) Determination

The Total phenolic content (TPC) was determined by the Folin–Ciocalteu method, as described by Singleton and Rossi Jr. (1965). The TPC of the propolis sample was determined by mixing 0.5 mL (1 mg/mL) of the sample in ethanol and 1 mL of Folin–Ciocalteu reagent; then the mixture was neutralized with 1 mL of 1% Na_2_CO_3_. After vigorous shaking, the mixture was incubated in the dark at room temperature for 2 h. Then, the absorbance of the sample was measured at 760 nm against a blank sample containing ethanol instead of the sample. The results were expressed as gallic acid equivalent (GAE) through a standard linear curve between 1 and 200 μg/mL (Aslan et al. 2024).

#### 2.3.2 Total Flavonoid Content (TFC) Determination

The total flavonoid content (TFC) was measured using the aluminum chloride colorimetric assay according to Zhishen et al. (1999). The TFC of the sample was determined by mixing 0.5 mL (1 mg/mL) of the sample in ethanol with 95% methanol, 0.5 mL of 1.0 M potassium acetate, 2.3 mL of distilled water, and 1.5 mL of 10% Al (NO_3_)_3_. After vortexing, the mixture was incubated at 25°C for 40 min, and the absorbance of the sample was recorded at 415 nm against a blank sample containing ethanol instead of the sample. The results were expressed as quercetin equivalent (QE) through a standard linear curve between 1 and 500 μg/mL (Aslan et al. 2024).

#### 2.3.3 Phenolics and Flavonoids Determination by LC–MS/MS

The determination of phenolics and flavonoid daisies by LC–MS/MS has been reported previously. Thirty‐five OAs and PCs were quantified in total by liquid chromatography (Agilent Technologies 1290 Infinity UHPLC chromatography, Palo Alto, USA), followed by electrospray ionization (ESI) MS–MS (Agilent 6460 mass spectrometer, Palo Alto, USA). UHPLC‐ESI‐MS/MS data were acquired and processed by MassHunter Qualitative Analysis B07 and MassHunter Quantitative Analysis B07 software (Agilent, USA). The LC–MS/MS method was previously developed and validated by our research group (Can et al. 2024; Güven et al. 2024).

##### 2.3.3.1 Chromatography and MS Analysis

UHPLC separation was performed using an Agilent 1290 Infinity system with an autosampler (G4226A), sampler thermostat (G1330B), quad pump (G4204A, 1200 bar), and thermostated column compartment (G1316A). A Zorbax SB‐C18 column (4.6 × 100 mm, 3.5 μm, USA) was used with a mobile phase of water (0.1% formic acid) and acetonitrile (0.1% formic acid) under a controlled column temperature of 30°C. For MS analysis, the multiple reaction monitoring (MRM) mode was used to quantify organic acids (OAs) and phenolics (PCs), optimizing precursor‐to‐fragment ion transitions. The scan range was 50–1300 m/z, with optimized collision energies. MS conditions included a drying gas (N_2_) at 350°C (12 L/min), nebulizing gas (N_2_) at 55 psi, sheath gas at 250°C (5 L/min), and a capillary voltage of 3.5 kV. Data acquisition was performed using the Agilent MassHunter Workstation (Can et al. 2024; Güven et al. 2024).

### 2.4 Determination of Antioxidant Activity

Six different methods with two different principles (radical scavenging and metal‐reducing capacity) were employed to determine the antioxidant activity of the propolis sample. The results were compared with the standard antioxidants, including ascorbic acid, BHA, BHT, tocopherol, and Trolox. Radical scavenging capacities were expressed as the mean IC_50_ value as the concentration that scavenges 50% of the total radical, calculated by the following formula derived from the trend of the graph:

$$y=\mathrm{ax}+b,{\mathrm{IC}}_{50}=\left(0.5-b\right)/a$$

Reducing capacities were expressed by the meanabsorbances±standard deviation obtained at relevant wavelengths in the assays. Each test was performed in triplicate.

#### 2.4.1 Radical Scavenging Assays

##### 2.4.1.1 DMPD Assay

1 mL of DMPD^•+^ solution in pH 5.3, 0.1 M acetate buffer, 0.2 mL of 0.05 M FeCl_3_, and 0.5 mL of standard antioxidants or the propolis sample in acetate buffer at different concentrations between 15 and 45 μg/mL. The total volume was adjusted to 2 mL, and the mixture was incubated dark at room temperature for 50 min. The absorbance of the sample was measured at 505 nm. The control reaction consisted of ethanol instead of the sample or standard antioxidants (Aslan et al. 2024; Karagecili, İzol, et al. 2023).

##### 2.4.1.2 DPPH Assay

The DPPH radical‐scavenging activity was measured according to the method of Brand‐Williams et al. (1995), with slight modifications in incubation time and solvent (Brand‐Williams et al. 1995). Preradicalization was performed for the preparation of the 0.1 mM DPPH radicals in ethanol by incubation for 16 h in the dark. The reaction mixture was prepared by the addition of 0.5 mL DPPH and 0.5 mL of varying concentrations of the sample (15–45 μg/mL) in ethanol and incubated in the dark at 30°C for 30 min. After incubation, the absorbances of the sample were measured at 517 nm wavelength. The control reaction consisted of ethanol instead of the sample or standard antioxidants (Aslan et al. 2024; Karagecili, İzol, et al. 2023).

##### 2.4.1.3 ABTS Assay

ABTS^+^ decolorization was measured as described by Re et al. (1999), with modifications (Re et al. 1999). Preradicalization of ABTS was also performed to prepare ABTS radicals by mixing 2 mM ABTS and 2.45 mM potassium thiosulfate in equal volumes and incubating in the dark for 6 h at room temperature. Before the test, the absorbance of the ABTS radicals at 734 nm was maintained around 1.0 with PBS buffer (0.1 M PBS, pH 7.4). The test was conducted by mixing 1 mL of ABTS radicals and 3 mL of varying concentrations of the samples (15–45 μg/mL), followed by absorbance measurement at 734 nm wavelength. The control reaction consisted of PBS buffer instead of the sample or standard antioxidants (Aslan et al. 2024).

#### 2.4.2 Reducing Assays

##### 2.4.2.1 Cupric Reducing Antioxidant Capacity (CUPRAC) Assay

Cupric‐reducing antioxidant capacity (CUPRAC) was assessed as described by Apak et al. (2004), employing neocuproine and acetate buffer at pH 7.0 (Apak et al. 2004). The test was conducted by mixing equal volumes of 10 mM of copper (II) chloride, 7.5 mM neocuproine, and 1.0 M ammonium acetate buffer (1.0 M pH 6.5), varying concentrations of the samples (15–45 μg/mL in acetate buffer) in a total reaction volume of 2 mL. Then, the mixture was incubated at 25°C for 30 min, and upon completion of incubation, the absorbances of the samples were recorded at 450 nm. The blank sample consisted of acetate buffer instead of the samples (Aslan et al. 2024).

##### 2.4.2.2 Fe^3+^‐Reducing Antioxidant Power (FRAP) Assay

The test was conducted by mixing 0.5 mL of varying concentrations of the samples (15–45 μg/mL in acetate buffer (0.3 M pH 3.6)), 2.25 mL of FRAP reagent, and 2.25 mL of 20 mM FeCl_3_ in a total 5 mL of reaction volume. The mixture was incubated at 37°C for 30 min, and then the absorbances were recorded at 593 nm. FRAP reagent was prepared as previously applied (Aslan et al. 2024). The blank sample consisted of acetate buffer instead of the samples.

##### 2.4.2.3 Fe^3+^‐Reducing Assay

Fe^3+^‐reducing power was determined by measuring the conversion of Fe^3+^ to Fe^2+^ in the presence of potassium ferricyanide, following the method of Oyaizu (1986). Total reducing power (FRAP) was evaluated following Benzie and Strain (1996) with slight modification (Benzie and Strain 1996). Equal volumes of PBS buffer (0.2 M, pH 6.6), 1% (w/w) potassium ferrocyanide were mixed with 0.75 mL of varying concentration (15–45 μg/mL) of the samples. After incubation at 50°C for 30 min, 1.25 mL of 10% trichloroacetic acid (w/w) and 0.25 mL of 0.1% Iron (III) chloride were added to the mixture. The mixtures were vortexed and analyzed at 700 nm wavelength. The blank sample consisted of PBS buffer instead of the samples (Aslan et al. 2024).

### 2.5 Determination of Enzyme Inhibition Properties

#### 2.5.1 α‐Glycosidase (AG) Inhibition Studies

α‐Glucosidase inhibitory activity was determined using p‐nitrophenyl‐α‐d‐glucopyranoside as substrate, following Tadera et al. (2006). α‐Glycosidase enzyme activity was measured kinetically using 5 mM 4‐nitrophenyl β‐D glucopyranoside substrate in the presence of PBS buffer (0.1 M, pH 6.9) for 5 min at 405 nm wavelength. The concentration of the tested sample was gradually increased in the reaction mixture until more than half of the activity of the enzyme was inhibited. The reference inhibitor was acarbose for the AG inhibition, and a control reaction was the activity measurement mixture without an inhibitory substance (Demir et al. 2019).

#### 2.5.2 Acetylcholinesterase (AChE) Inhibition Studies

Acetylcholinesterase and butyrylcholinesterase activity was assayed colorimetrically according to Ellman et al. (1961) using DTNB and acetylthiocholine iodide (Ellman et al. 1961). AChE inhibition was measured kinetically using 50 μL of 10 mM DTNB and 10 mM acetylcholine iodide as substrate in the presence of 100 μL Tris.HCl buffer (1 M, pH 8.0) for 5 min at 412 nm (Aslan et al. 2024). The concentration of the tested sample was gradually increased in the reaction mixture until more than half of the activity of the enzyme was inhibited. The reference inhibitor was donepezil for the AChE inhibition, and a control reaction was the activity measurement mixture without an inhibitory substance (Aslan et al. 2024).

#### 2.5.3 Human Carbonic Anhydrase I (hCA I) and Human Carbonic Anhydrase II (hCA II) Enzymes

Carbonic anhydrase esterase activity was measured spectrophotometrically using p‐nitrophenyl acetate as substrate, following the method of Wilbur and Anderson (1948) by monitoring the increase in absorbance at 348 nm (Wilbur and Anderson 1948). Enzyme inhibition properties of hCA I and hCA II were determined over their kinetic esterase activities at 348 nm. For this, 360 μL of 0.07 mM p‐nitrophenyl acetate, 400 μL of Tris‐SO_4_ buffer (0.05 M pH 7.4), and 20 μL of the enzyme solution were mixed in a total volume of 1 mL. The concentration of the tested sample was gradually increased in the reaction mixture until more than half of the activity of the enzyme was inhibited. The reference inhibitor was tacrine for the hCA I and hCA II inhibition, and a control reaction was the activity measurement mixture without an inhibitory substance.

### 2.6 Statistical Analysis

Each experiment is repeated three times. The results are given as mean ± SD. In the two‐way ANOVA, significant differences were considered to have a value of *p* < 0.05. All data were processed, and graphs were created using GraphPad Prism 8.0.2. The IC_50_ value refers in this study to the concentration of an inhibitor needed to inhibit an enzyme activity response by 50%. The calculation of the IC_50_ values was performed using the GraphPad Prism 8.4.0 non‐linear regression‐[inhibitor]‐normalized response (y values 100 down to 0) model.

## 3 Results

### 3.1 Phytochemistry of the Siran Propolis

The total flavonoid content of the extracts was performed separately since flavonoids are a group of polyphenolic compounds and are separable from polyphenols by their C‐skeleton number (Gulçin 2020). Most flavonoids contain only 15 C‐skeletons, whereas this number may differ in polyphenols. Therefore, it is expected to have higher TPC content than TFC content. The TPC analysis was performed using the Folin–Ciocalteu method based on the formation of reduced molybdenum complex in the presence of a phenolic structure (Gulçin 2020). On the other hand, the TFC analysis relied on Al^3+^‐flavonoid complex formation that gives absorption maxima at 415 nm (Gulçin 2020). Based on the results obtained from the TPC analysis, 337.78 ± 0.01 mg GAE/g of extracts were determined in the sample (*p* < 0.05). The TFC of the propolis was determined as 91.50 ± 4.84 mg QE/g of propolis sample (*p* < 0.05).

**FIGURE 1:**
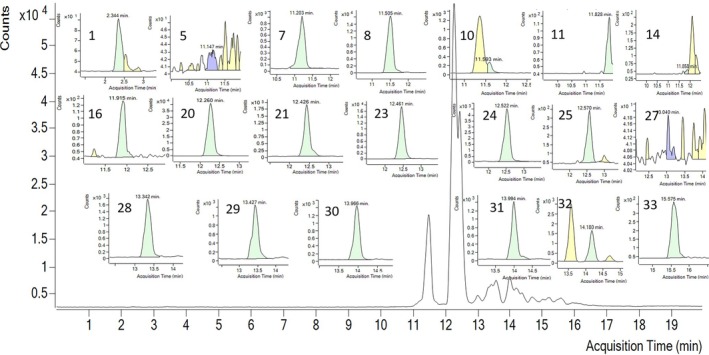
LC–MS/MS chromatogram of Siran Propolis; Inset: MRM resolutions of the metabolites, red numbers refer to each standard in Table 1.

LC–MS/MS analysis of the propolis sample was performed against 35 different phenolic compounds commonly found in plants and plant‐derived natural products (Table 1). Twenty of them were determined in the extracts: ferulic acid (40396.72 ng/g of extract), p‐coumaric acid (8857.88 ng/g), vanillin (7273.81 ng/g), vanillic acid (3572.81 ng/g), and caffeic acid (3279.34 ng/g) were concluded as major compounds. In addition, quinic acid, cyanidin‐3‐o‐glucoside, keracyanin chloride, peonidin‐3‐o‐glucoside, 4‐OH‐benzoic acid, naringin, taxifolin, resveratrol, luteolin, quercetin, apigenin, naringenin, isorhamnetin, and galangin were determined in the extract. Details of the quantitative LC–MS/MS result and the chromatogram can be found in Table 1 and Figure 1.

**TABLE 1:** Quantitative LC–MS/MS results of Siran Propolis ethanolic extracts.

	Compound	Acquisition time	Response	Concentration (ng/g of extract)
1	Quinic Acid	2.34	472	149.77
2	Fumaric Acid	3.70	34	<LOD
3	Gallic Acid	5.48	64	<LOD
4	Pyrogallol	6.36	3	<LOD
5	Cyanidin‐3‐o‐glucoside	11.86	113	36.21
6	Chlorogenic Acid	10.82	268	<LOD
7	Keracyanin Chloride	11.83	731	223.30
8	Peonidin‐3‐o‐glucoside	11.15	33	4.80
9	Catechin	11.08	50	<LOD
10	4‐OH‐Benzoic Acid	11.20	10,607	635.01
11	Caffeic Acid	11.51	182,362	3279.34
12	Epicatechin	11.23	18	<LOD
13	Epigallocatechin Gallate	11.38	5	<LOD
14	Vanillic Acid	11.59	1758	3572.81
15	Vitexin	11.73	324	<LOD
16	Naringin	11.92	814	42.20
17	Syringic Acid	11.75	5	<LOD
18	Hesperidin	12.00	28	<LOD
19	Ellagic Acid	12.05	32	<LOD
20	p‐Coumaric Acid	12.26	447,279	8857.89
21	Taxifolin	12.43	18,841	343.06
22	Sinapic Acid	12.39	6	<LOD
23	Rosmarinic Acid	12.57	2854	714.11
24	Ferulic Acid	12.46	175,934	40396.72
25	Vanillin	12.52	50,587	7273.81
26	Myricetin	12.74	119	<LOD
27	Resveratrol	13.04	7	2.95
28	Luteolin	13.34	19,330	111.85
29	Quercetin	13.43	14,207	287.56
30	Apigenin	13.97	14,682	66.36
31	Naringenin	13.99	14,105	230.39
32	Isorhamnetin	14.18	16,033	15.41
33	Galangin	15.58	3890	155.87
34	Curcumin	15.77	1	<LOD
35	Chrysin	15.50	2090	<LOD

Abbreviation: <LOD, limit of detection.

### 3.2 Antioxidant Profile of Siran Propolis

**FIGURE 2:**
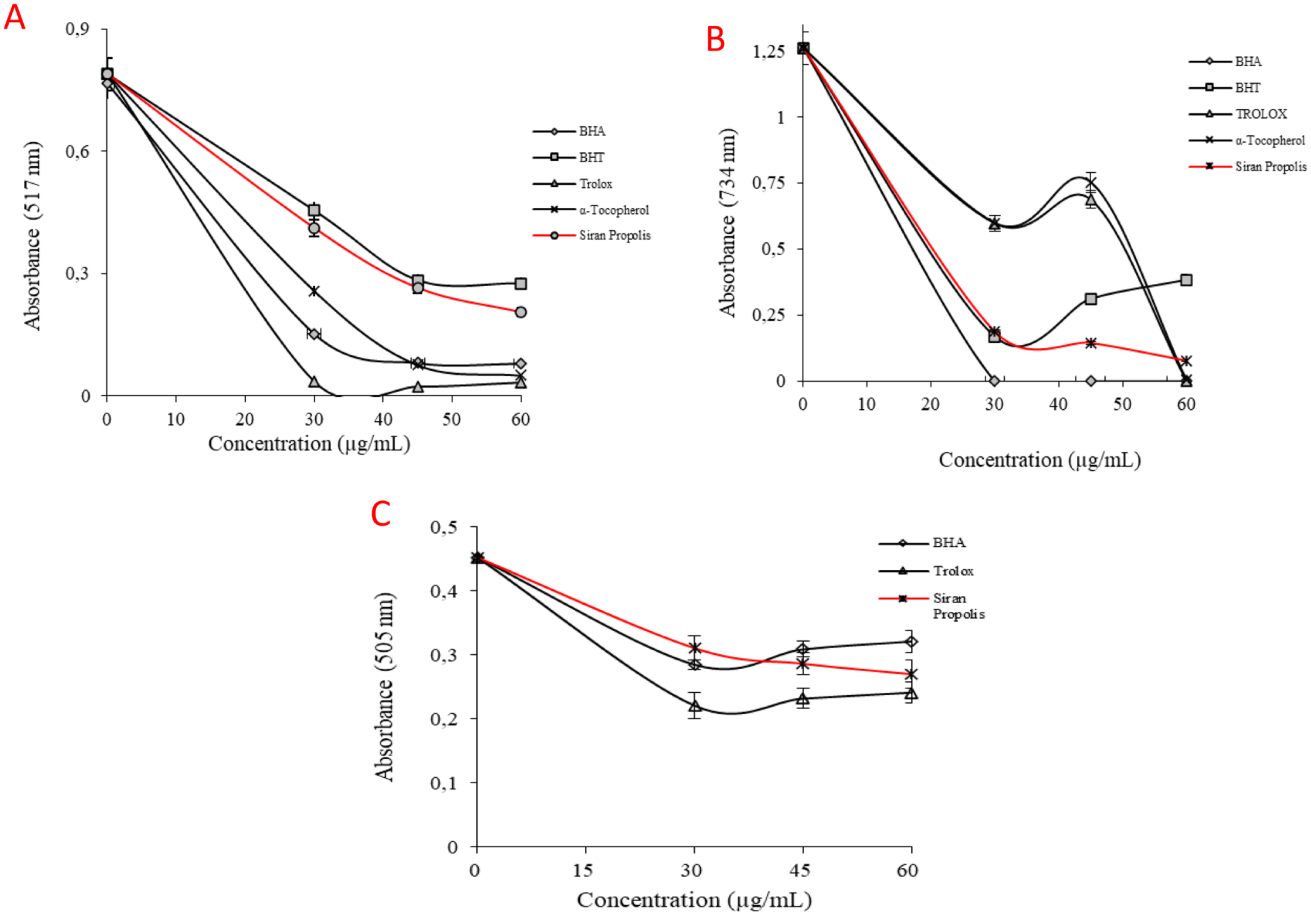
Radical scavenging results of ethanolic Siran propolis. (A) DPPH^·^ scavenging assay (B) ABTS^·+^ scavenging assay (C) DMPD^·+^ scavenging assay (*p* < 0.05).

The antioxidant properties of the Siran propolis were investigated through radical scavenging assays and reducing capacity tests. According to the ^·^DPPH test results (Figure 2A), IC_50_ values were calculated as 30.14 ± 0.27 μg/mL for ethanolic propolis, 11.95 ± 0.21 μg/mL for Trolox, 14.44 ± 0.08 μg/mL for α‐tocopherol, 17.33 ± 0.08 μg/mL for BHA, and 36.48 ± 0.28 μg/mL for BHT (*p* < 0.05). The efficiency in the DPPH radical scavenging property of propolis was evaluated as higher than the standard antioxidant, BHT. On the other hand, the IC_50_ value in the ABTS assay was calculated to be 272.44 ± 17.70 μg/mL, which was significantly higher than that of the standard antioxidant, rendering the propolis extract inefficient in ABTS^·+^ scavenging (Table 2, Figure 2B). Lastly, according to the DMPD^·+^ results, the IC_50_ value of the ethanolic propolis was calculated to be 77.02 ± 2.80 μg/mL, while the IC_50_ values for BHA and Trolox were 115.53 ± 3.10 μg/mL and 63.01 ± 2.32 μg/mL, respectively (*p* < 0.05) (Figure 2C).

**TABLE 2:** Radical scavenging activity results of Siran propolis.

Antioxidants	DPPH^·^ scavenging assay		ABTS· + scavenging assay		DMPD^·+^ scavenging assays	
	(IC_50_)	*r* ^2^	(IC_50_)	*r* ^2^	(IC_50_)	*r* ^2^
BHA^a^	17.33 ± 0.08	0.9865	24.17 ± 1.34	0.9865	115.53 ± 3.10	0.9664
BHT^a^	36.48 ± 0.28	0.9815	34.66 ± 2.17	0.9685	—	—
α‐Tocopherol^a^	14.44 ± 0.08	0.9877	35.39 ± 3.71	0.9287	—	—
Trolox^a^	11.95 ± 0.21	0.9838	33.81 ± 1.18	0.9732	63.01 ± 2.32	0.9835
Siran Propolis	30.14 ± 0.27	0.9975	272.44 ± 17.70	0.9342	77.02 ± 2.80	0.9514

^a^ The results were compared with the standard antioxidants, including ascorbic acid, BHA, BHT, tocopherol, and Trolox (*p* < 0.05).

**FIGURE 3:**
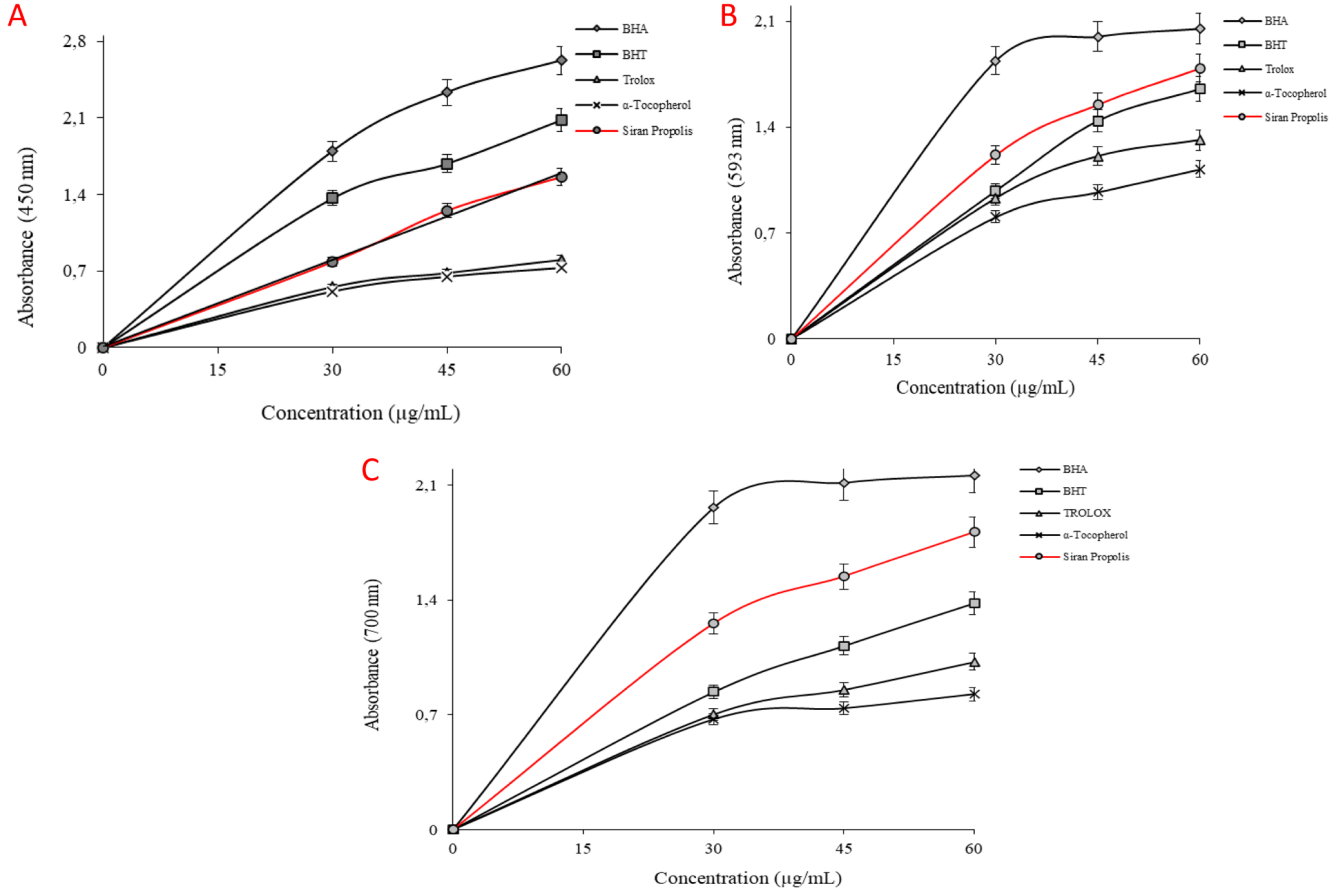
Reducing results of ethanolic Siran propolis. (A) CUPRAC assay, (B) FRAP assay, (C) Fe^*3*+^‐reducing assay (*p* < 0.05).

Reducing capacity tests in this study include CUPRAC, FRAP, and Fe^3+^‐reducing assays. Reducing antioxidant power is based on the principle of an increase in the absorbance of the reaction mixtures. An increase in absorbance indicates an increase in antioxidant activity (Gulçin 2020). The results obtained from the CUPRAC assay can be ordered in descending order as BHA > BHT > Siran propolis > Trolox > α‐Tocopherol. This order in FRAP and Fe^3+^‐reducing assay was parallel to each other: BHA > Siran propolis > BHT > Trolox > α‐Tocopherol (*p* < 0.05). Detailed absorbance values are shown in Table 2, and the graphical representation of the results can be found in Figure 3A–C (Table 3).

**TABLE 3:** Reducing capacity test results of Siran propolis.

Antioxidants	CUPRAC assay		FRAP assay		Fe^3+^ reducing assay	
	λ_450_	*r* ^2^	λ_595_	*r* ^2^	λ_700_	*r* ^2^
BHA^a^	2.62 ± 0.03	0.9558	2.05 ± 0.01	0.9401	2.16 ± 0.06	0.9320
BHT^a^	2.08 ± 0.09	0.9715	1.65 ± 0.02	0.9810	1.38 ± 0.10	0.9860
α‐Tocopherol^a^	0.73 ± 0.02	0.9485	1.12 ± 0.04	0.9430	0.83 ± 0.02	0.9899
Trolox^a^	0.80 ± 0.03	0.9573	1.31 ± 0.02	0.9430	1.02 ± 0.01	0.9590
Siran Propolis	1.56 ± 0.06	0.9967	1.79 ± 0.01	0.9430	1.81 ± 0.09	0.9560

^a^ The results were compared with the standard antioxidants, including ascorbic acid, BHA, BHT, tocopherol, and Trolox (*p* < 0.05).

### 3.3 Enzyme Inhibition Profile of Siran Propolis

**FIGURE 4:**
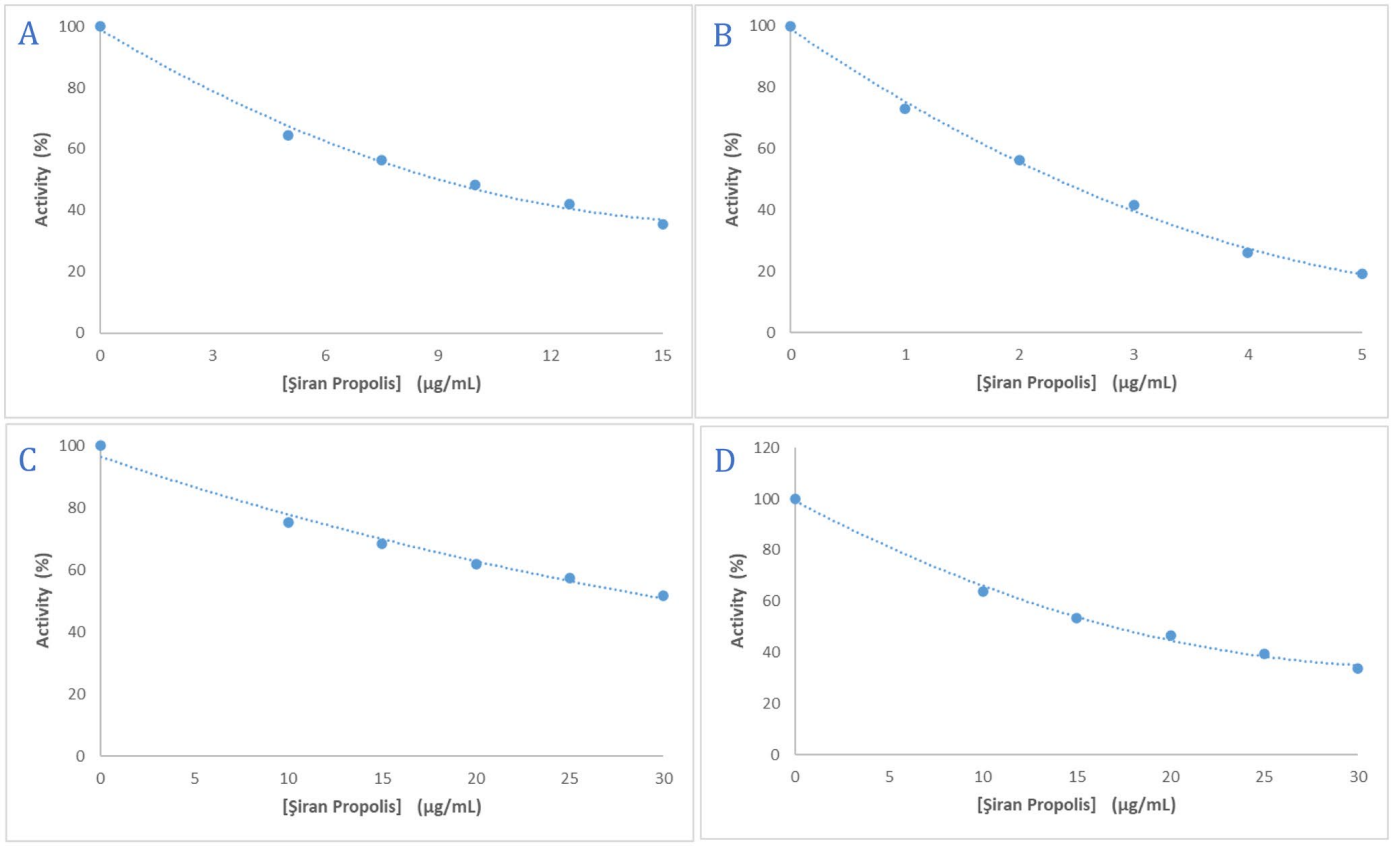
Percentage of enzyme inhibition results of ethanolic propolis extracts: (A) α‐glycosidase enzyme, (B) AChE enzyme, (C) hCA I isoenzyme, and (D) hCA II isoenzyme.

The α‐glycosidase enzyme inhibition of Siran propolis was assessed using 4‐nitrophenyl β‐D‐glucopyranoside as a substrate, where the hydrolysis product, p‐nitrophenol, was measured at 405 nm (Demir et al. 2019). The ethanolic propolis extract exhibited 64.5% inhibition at 5 μg/mL (Figure 4A) with an IC_50_ value of 9.71 μg/mL.

The inhibition of hCA I and hCA II enzymes was determined based on their esterase activity, with the reaction product measured at 348 nm (Demir 2024). The ethanolic propolis extract inhibited hCA I and hCA II activity by 51.69% and 70.33%, respectively, compared to 65.60% and 70.13% inhibition by the standard inhibitor acetazolamide (AZA) (Figure 4C,D). The IC_50_ values of propolis extract were determined as 30.07 μg/mL for hCA I and 18.45 μg/mL for hCA II, while AZA exhibited IC_50_ values of 15.83 μg/mL and 8.44 μg/mL, respectively.

The AChE enzyme inhibition was determined using 5,5′‐dithiobis‐2‐nitrobenzoic acid (DTNB, Ellman's reagent) as a chromogen and recording the level of cholinesterase activity as an increase of absorbance at 412 nm (Gümüş et al. 2022; Yaşar et al. 2021). The ethanolic propolis extract inhibited 81.80% of the total activity when 5 μg/mL of the extract was used (Figure 4B). The ethanolic propolis extract exhibited strong AChE inhibition, achieving 81.80% inhibition at 5 μg/mL (Figure 4B) with an IC_50_ of 2.15 μg/mL, compared to 8.82 μg/mL for donepezil, indicating a fourfold higher efficacy (Table 4).

**TABLE 4:** IC_50_ values (μg/mL) calculated for ethanolic propolis over metabolic enzymes.

Samples	α‐Glycosidase		AChE		hCA I		hCA II	
	IC_50_	*r* ^2^	IC_50_	*r* ^2^	IC_50_	*r* ^2^	IC_50_	*r* ^2^
Propolis	9.71	0.9897	2.15	0.9950	30.07	0.9869	18.35	0.9940
Standards^a^	9.43	0.9995	8.82	0.9836	15.83	0.9836	8.44	0.9930

^a^ Acarbose was employed as a positive control for α‐amylase, and donepezil for AChE. The IC_50_ value for Acetazolamide for carbonic anhydrase isoenzymes.

## 4 Discussion

Phytochemical analysis of the propolis was performed by analyzing the TPC and TFC content of the sample and LC–MS/MS analysis. Previous reports of propolis collected from Türkiye yielded lower TPC and TFC content; for instance, Berdav propolis yielded 53 mg GAE/g of propolis sample and 170 mg QE/g of the sample (Karagecili, Yılmaz, et al. 2023). Similarly, Erzurum propolis yielded 124.30 (GAE)/g of propolis extracts, while 8.15 QE/g of sample (Gülçin et al. 2010). A comprehensive study investigating the propolis extracts collected from the different regions of Türkiye, from north to south and east to west, has revealed that among the samples, the propolis from the north region (Ordu Province) has more TPC and TFC content than the others, involving 492.30 ± 5.80 mg GAE/g of extracts and 96.10 ± 2.10 QE/g of sample (İzol et al. 2024). A study conducted on the chemical composition of five different propolis samples obtained from Morocco and Palestine yielded TPC contents between 74.71 ± 0.89 and 148 ± 1.31 mg GAE/g of extracts and TFC contents between 26.97 ± 2.44 and 118 ± 1.92 QE/g of samples. The higher results belonged to Moroccan propolis (Touzani et al. 2021). On the other hand, Indonesian Propolis has been reported to constitute the TPC and the TFC as 1037.31 ± 24.10 μg GAE/mL and 374.02 ± 3.36 μg QE/mL, respectively, having the richest propolis so far (Tandean et al. 2024). However, the differences in the expressed results are significant.

Despite the characteristic phytochemical similarities of propolis within the same geographical zone, region‐specific uniqueness and variation in chemical composition have also been observed in the studies (Kasote et al. 2022). This phenomenon has been related to region‐specific flora. A study conducted for the propolis collected from the Ordu (black sea climate) has determined 33 different phenolics and flavonoids among 56 phytochemical standards. p‐Coumaric acid 66.454 (mg analyte/g extract), caffeic acid 47.779 (mg analyte/g extract), ferulic acid 7.052 (mg analyte/g extract), isoquercitrin 2.855 (mg analytes/g extract), quercetin 8.673 (mg analytes/g extract), and naringenin 8.468 (mg analytes/g extract) were detected in the extracts (İzol et al. 2024). A conclusion made from a similar study investigating the phytochemistry of propolis collected from different regions of Türkiye revealed 4 flavonoids: quercetin, apigenin, pinocembrin, and 6 phenolic acids: caffeic acid, p‐coumaric acid, trans‐ferulic acid, protocatechuic acid, trans‐cinnamic acid, caffeic acid phenethyl ester (CAPE) components have been detected as mg/g, in different ratios in propolis samples collected from different regions of Türkiye (Özkök et al. 2021). The results obtained from this study were consistent with the characteristics of the Turkish propolis, and temperate zone and Mediterranean characteristics were concluded (Kasote et al. 2022).

Radical scavenging tests, including DPPH^·^, ABTS^·+^, and DMPD^·+^ assays, are commonly used to evaluate antioxidant capacity. These chemical assays measure the ability of compounds to neutralize synthetic free radicals through various radical‐generating systems and oxidation detection methods. Spectrophotometric techniques based on DPPH^·^, ABTS^·+^, DMPD^·+^, and O_2_^·−^ radicals are widely employed for this purpose. When antioxidants interact with these radicals, they cause a degree of decolorization, indicating their scavenging activity and the reversal of radical formation (Gulçin 2020). The previous study determined IC_50_ values in DPPH and ABTS radical scavenging assays as 8.88 ± 0.84 μg/mL and 4.59 ± 0.80 μg/mL for Ordu propolis, 9.24 ± 0.67 μg/mL and 5.50 ± 0.14 μg/mL for Igdır Propolis, 10.83 ± 0.71 μg/mL and 7.79 ± 0.59 μg/mL for Manisa Propolis, and 15.75 ± 0.63 μg/mL and 13.08 ± 0.35 μg/mL for Mus Propolis, 20.55 μg/mL and 8.157 μg/mL for Berdav Propolis, and 16.70 ± 0.09 μg/mL and 8.01 ± 0.05 μg/mL for Bitlis Propolis (İzol et al. 2024; İzol and Turhan 2024; Karagecili, Yılmaz, et al. 2023). A comparison of the findings of the present study showed that ethanolic Siran propolis yielded lower efficiency at radical scavenging among the local studies. Only the DMPD^·+^ scavenging capacity was found to be higher than Berdav Propolis (86.64 μg/mL) (Karagecili, Yılmaz, et al. 2023). Reducing capacity results obtained from the present study were found to be remarkably higher than the local studies conducted with Ordu, Igdır, Mus, Manisa, Berdav, Bitlis, and Erzurum Propolis (Gülçin et al. 2010; İzol et al. 2024; İzol and Turhan 2024; Karagecili, Yılmaz, et al. 2023). Since the expression of the results differs in the global studies as well as the absence of comparison with standard antioxidants, it will not be proper to compare the results; however, among the studies, Algerian propolis showed a higher reducing capacity than the standard antioxidant, BHT. The reducing assays (CUPRAC, FRAP, Fe^3+^‐reducing) measure the electron‐donating ability of antioxidants, which reflects their capacity to reduce oxidized intermediates. Propolis demonstrated a strong reducing power, surpassing Trolox and α‐tocopherol.

The radical scavenging assays (DPPH^·^, ABTS^·+^, DMPD^·+^) assess the ability to neutralize free radicals through hydrogen atom transfer (HAT) or single electron transfer (SET) mechanisms (Gulçin 2020). Propolis performed better in DPPH· and DMPD^·+^ but was weak in ABTS·+ scavenging. The higher reducing capacity does not always correlate with better radical scavenging activity. While propolis showed strong reducing power, its efficiency varied across radical scavenging assays, suggesting differences in reaction mechanisms with different radicals (Gulçin 2020). The high ferulic acid and p‐coumaric acid content explains the strong reducing capacity of propolis, as these compounds effectively donate electrons. The presence of flavonoids (quercetin, luteolin, apigenin, resveratrol, etc.) contributes significantly to both radical scavenging and reducing power, as these compounds exhibit dual antioxidant mechanisms (Caporali et al. 2022; Günal‐Köroğlu et al. 2024; Karaoğlan and Koca 2020; Zhou et al. 2021). The lower ABTS^·+^ scavenging efficiency suggests that certain phenolic compounds in the extract may not interact efficiently with this radical, indicating selectivity in antioxidant mechanisms.

α‐Glycosidase and α‐amylase enzymes play a crucial role in carbohydrate digestion and absorption in the gastrointestinal system, and their inhibition is a key strategy for managing hyperglycemia. Commercial anti‐diabetic drugs such as acarbose, miglitol, and voglibose act via α‐glycosidase inhibition; however, they are often associated with adverse effects, including flatulence, severe gastrointestinal discomfort, and allergic reactions, necessitating the search for alternative inhibitors with fewer side effects (Kashtoh and Baek 2022). Propolis extracts have been investigated previously for their inhibitory activity on the α‐glycosidase enzyme (Gercek et al. 2024; İzol and Turhan 2024; Kashtoh and Baek 2022). Berdav propolis exhibited an IC_50_ value of 3.70 μg/mL, while Bitlis propolis showed an IC_50_ of 5.72 μg/mL (İzol and Turhan 2024; Karagecili, Yılmaz, et al. 2023). Propolis from Khuzestan and Mazandaran had IC_50_ values of 0.066 mg/mL and 2.160 μg/mL, respectively (Assaie Ardakani et al. 2024). The IC_50_ value determined in the present study was notably lower than those reported in previous studies, suggesting a stronger inhibitory effect. This enhanced inhibition may be attributed to the higher ferulic acid content in the extract, as ferulic acid has been shown to suppress the activity of porcine pancreatic α‐amylase and α‐glycosidase with IC_50_ values of 0.622 and 0.866 mg/mL, respectively, via a non‐competitive inhibition mechanism (Zheng et al. 2020).

Various endogenous and external factors, such as pH, temperature, and zinc ion concentration, influence the enzymatic activity of hCA I and hCA II. Additionally, numerous studies have explored selective CA inhibitors for their potential therapeutic applications. Since the clinical regulation of hCA activity through inhibitors has been a well‐established therapeutic approach for several diseases, including hypertension, glaucoma, hyperthyroidism, hypoglycemia, and, more recently, cancer, it remains a key focus in drug development (Demir 2024). Inhibition of hCA II, in particular, has been widely explored in the treatment of glaucoma, where reducing aqueous humor production lowers intraocular pressure (Kumar et al. 2021). Moreover, both hCA I and II inhibitors have shown promise in managing epilepsy, diuretic resistance, and metabolic disorders, including obesity and type 2 diabetes, by influencing CO_2_/bicarbonate balance and insulin sensitivity (Kumar et al. 2021). However, clinically used inhibitors of CA (acetazolamide, brinzolamide, dorzolamide, etc.) are not selective, causing undesirable side effects (Demir 2024). Previous studies reported Mus propolis as an hCA II inhibitor with an IC_50_ of 8.60 μg/mL, comparable to AZA (IC_50_ = 8.98 μg/mL). Similarly, two types of Ankara propolis inhibited hCA I and hCA II with IC_50_ values of 1.273 ± 0.352 μg/mL and 0.486 ± 0.032 μg/mL (Propolis 1), and 1.374 ± 0.066 μg/mL and 0.612 ± 0.010 μg/mL (Propolis 2), respectively (Gercek et al. 2024; Karagecili, Yılmaz, et al. 2023). The IC_50_ values of AZA in that study (0.011 ± 0.002 μg/mL for hCA I and 0.004 ± 0.001 μg/mL for hCA II) were consistent with the current findings. Additionally, caffeic acid phenethyl ester (CAPE), a bioactive component of propolis, has been reported to moderately inhibit hCA I, hCA II, hCA IX, and hCA XII (Gülçin et al. 2016). Although this study did not assess CAPE content, the presence of caffeic acid, ferulic acid, and quinic acid suggests a possible contribution to the hCA inhibitory activity of the ethanolic propolis extract. The observed inhibitory effects of Şiran propolis against hCA I and II in our study suggest potential therapeutic value, especially as natural products may offer alternative scaffolds with fewer side effects than current sulfonamide‐based drugs (Demir 2024).

Normal cholinergic signal transduction related to learning and memory depends on acetylcholine levels. People with Alzheimer's disease (AD) could develop severe ACh deficiency. At present, there are many drug therapies targeting AChE, which is the most common therapeutic target globally (Chen et al. 2022). Therefore, AChE inhibitors (AChEIs) like donepezil, rivastigmine, and galantamine, which were approved over two decades ago, remain the mainstay of AD treatment in clinical management. Studies on other propolis samples reported varying IC_50_ values, such as 5.17 μg/mL for Bitlis propolis, 3.4 μg/mL for Berdav propolis, and 16.98–54.36 μg/mL for Ankara propolis, compared to tacrine (IC_50_ = 8.15–8.28 μg/mL) (Gercek et al. 2024; İzol and Turhan 2024; Karagecili, Yılmaz, et al. 2023). Algerian propolis exhibited IC_50_ values between 71.29 and 180.80 μg/mL, while galantamine had an IC_50_ of 6.27 μg/mL (Boulechfar et al. 2022). A study on 20 propolis samples from Korea, Australia, Brazil, and China reported IC_50_ values ranging from 15.6 to 327.3 μg/mL, whereas tacrine had an IC_50_ of 1.2 μg/mL (Wang et al. 2016). These comparisons suggest that the propolis extract investigated in this study demonstrated notably higher AChE inhibitory activity than many previously reported propolis samples.

## 5 Conclusion

The antioxidant activity of the ethanolic propolis extract, as demonstrated by reducing power and radical scavenging assays, can be attributed to its diverse phenolic composition. LC–MS/MS analysis identified ferulic acid, p‐coumaric acid, vanillin, vanillic acid, and caffeic acid as the major constituents, along with flavonoids such as quercetin, luteolin, and taxifolin, which are known for their strong electron‐donating and radical‐neutralizing properties. These findings highlight the significant role of phenolic compounds in the antioxidant potential of propolis and support its potential use as a natural antioxidant source.

The ethanolic propolis extract demonstrated notable enzyme inhibition, particularly against AChE, hCA I, hCA II, and α‐glycosidase, indicating its potential therapeutic applications. The extract demonstrated a fourfold stronger AChE inhibition compared to donepezil, suggesting its potential for the treatment of neurodegenerative diseases. Although less potent than acetazolamide, the inhibition of hCA I and hCA II highlights its possible use in treating metabolic and ophthalmic disorders. The α‐glycosidase inhibition results also indicate its potential in managing postprandial hyperglycemia. The observed effects may be attributed to bioactive compounds such as caffeic and ferulic acids, which warrant further investigation for their clinical applications.

## Author Contributions

**Neslihan Balci:** conceptualization (equal), formal analysis (supporting), resources (equal). **Kubra Aslan:** conceptualization (equal), formal analysis (equal), investigation (equal), methodology (equal), resources (equal), writing – original draft (equal), writing – review and editing (equal). **Adem Erturk:** formal analysis (equal), investigation (equal), methodology (equal), resources (equal), writing – review and editing (equal). **Ilhami Gulcin:** conceptualization (equal), investigation (equal), methodology (equal), supervision (equal), writing – original draft (equal).

## Conflicts of Interest

The authors declare no conflicts of interest.

## Data Availability

The data that support the findings of this study are available from the corresponding author upon reasonable request.
